# Performance of multi-trait and single-trait genomic selection for grain Fe and Zn concentrations in sorghum under different breeding constraints

**DOI:** 10.1038/s41598-025-29176-y

**Published:** 2025-12-29

**Authors:** Ephrem Habyarimana, Sonal Chavan, Chaitra Hugar, Suvarna, Niranjan Ravindra Thakur, Marco Lopez-Cruz, Jieqin Li, Pradeep Ruperao

**Affiliations:** 1 International Crops Research Institute for the Semi-Arid Tropics, Patancheru, 502324 India; 2 University of Agricultural Sciences, Raichur, 584 104 Karnataka India; 3 Department of Epidemiology and Biostatistics, Michigan State University, East Lansing, MI 48824 USA; 4 College of Agriculture, Anhui Science and Technology University, Fengyang, 233100 China

**Keywords:** Micronutrients biofortification, Genomic selection, Multi-trait GBLUP, Prediction accuracy, Sorghum bicolor, Genetics, Plant sciences

## Abstract

Sorghum biofortification is a cost-effective approach to solving the issue of micronutrient deficiencies in human diets. Research programs face challenges, e.g., phenotyping time, cost, and accuracy in evaluating breeding populations across years and environments, which can be addressed through genomic selection (GS). The present GS work on sorghum grain Fe and Zn contents revealed that the multi-trait genomic best linear unbiased prediction (GBLUP) model (MT-GBLUP) consistently outperformed single-trait GBLUP (ST-GBLUP) in terms of prediction accuracy (PA) under different breeding resource-constrained scenarios. The PA gain by MT-GBLUP for Fe (0.274) and Zn (0.183) was greater when information was borrowed from auxiliary agronomic traits evaluated in a few locations than when only highly correlated target traits (Fe and Zn) were evaluated in more years and locations (PA gain ≤ 0.005). These results suggest that easily scorable non-target traits can inform and improve MT-GBLUP prediction accuracy for the genomic estimated breeding values of the target traits, thereby significantly saving multi-environment testing resources and potentially boosting genetic gain per unit time and cost.

## Introduction

Global food production has increased significantly, yet billions still face hunger and malnutrition. Malnutrition includes nutritional deficiencies and excesses, affecting 691 to 783 million people worldwide, with over half of children under five and two-thirds of adult women suffering from micronutrient deficiencies, known as “hidden hunger"^1^. Essential micronutrients like iodine, vitamin A, iron, and zinc are critical for health, especially in low and middle-income countries^2^. Anaemia, often linked to low dietary iron bioavailability^3^, and zinc deficiency are major health threats, contributing to various disorders and being the fifth highest risk factor for diseases^4^. Biofortification of food grains is vital for enhancing public health and economic stability^5^. Climate change could increase hunger and malnutrition risk by 20% by 2050, worsening child mortality rates^6^. In this context, sorghum, a C4 photosynthetic crop, is vital for food security due to its resilience to extreme weather^7^. It serves multiple roles, including staple grain and livestock feed, supporting over 500 million people in semi-arid regions^8^. Breeding dual-purpose sorghum cultivars that provide both grain and fodder can enhance nutrient density and climate resilience, benefiting local economies^9^.

Conventional breeding programs rely on phenotypic data from individuals and their relatives to select for important quantitative traits^10^. However, slow breeding cycles limit genetic gains, as these traits are often influenced by numerous small-effect loci with low heritability. Genomic selection (GS) addresses these issues by utilizing genomic information to rapidly identify superior genotypes, thereby accelerating breeding cycles^11,12^. GS improves precision in breeding value predictions by incorporating data from all available markers, thereby overcoming the limitations of marker-assisted selection (MAS) and enhancing genetic gains crucial for crop improvement programs^13,14^.

**Fig. 1:**
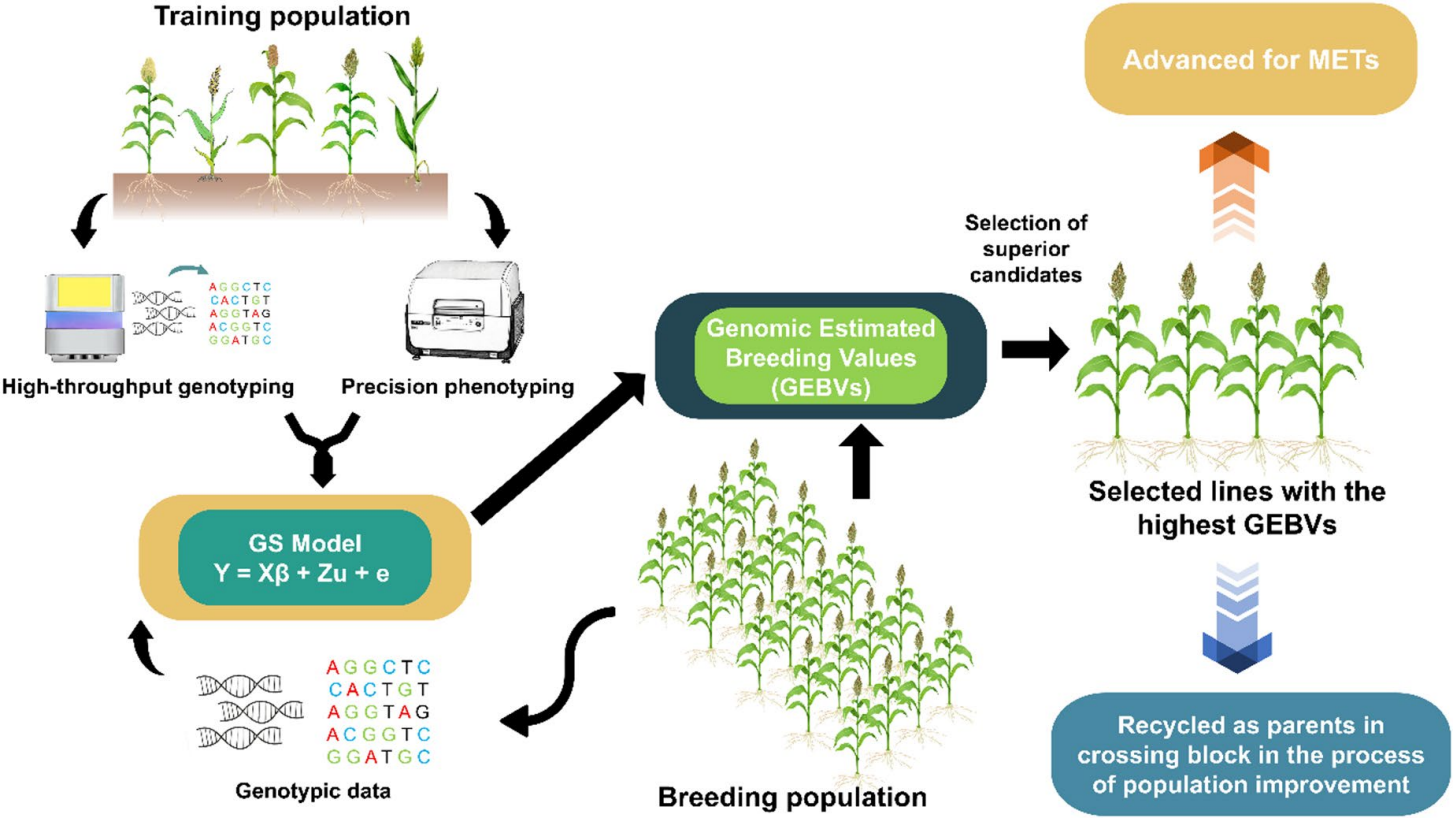
Diagrammatic representation of the genomic selection as a breeding approach.

Genomic selection represents a pivotal advancement in breeding methodologies, employing statistical models to estimate marker effects or model genomic relationships in a training population using phenotypic and genotypic data, which is then used to predict the genetic value of individuals only based on genomic information^10,15^. The marker effects are used to produce genomic-estimated breeding values (GEBVs) for genotyped lines within the breeding population, and the GEBVs are used to select superior candidates^10,12^ (Fig. 1). GS has been successfully implemented in various animal husbandry and plant breeding programs. However, its application is limited in sorghum when compared to other cereal crops such as maize, wheat, and rice^16,17^.

Pedigree selection, mass selection, single-seed selection, backcross breeding, and hybrid breeding are key methods in plant breeding that can significantly benefit from genomic selection (GS). These strategies aim to create new and improved plant varieties according to factors like reproductive mode, desired traits, and resource availability^18^. GS enhances traditional pedigree selection by employing genome-wide markers, increasing the accuracy of predicting breeding values for complex traits. Unlike approaches relying solely on family history, GS computes a genomic breeding value (GEBV) using DNA markers. A training population with both phenotypic and genotypic data develops a model to estimate GEBV for young or unphenotyped individuals based on their marker profiles. This process allows breeders to identify superior genotypes earlier and more accurately, reducing generation intervals and increasing genetic gain compared to conventional methods^19^.

Additionally, GS surpasses traditional mass selection techniques. For example, it has been shown to yield a 20% increase in maize production and a 21% improvement in selection indices for common buckwheat. This effectiveness is particularly pronounced for traits that are difficult to measure or influenced by environmental conditions, enabling breeders to predict values without waiting for maturation or progeny testing^20^. Backcross breeding benefits from GS by accelerating the recovery of the recurrent parent’s genome while introducing traits from a donor parent. By utilizing genomic data, GS allows for the earlier selection of elite individuals, effectively halving the breeding cycle time and enhancing genetic gain by up to 50%^21^. In hybrid breeding, GS aids in quickly developing hybrid varieties by predicting performance based on parental genomic data. It constructs statistical models to estimate combining abilities and overall hybrid performance^22^. Moreover, GS can be integrated into the Single-Seed Descent (SSD) method, facilitating the identification of superior individuals in early generations without extensive phenotyping. This approach streamlines breeding cycles and accelerates genetic gains^19,23^.

Single-trait Genomic Best Linear Unbiased Prediction (ST-GBLUP) has traditionally been used for predicting genetic values for individual traits. In contrast, multi-trait Genomic Best Linear Unbiased Prediction (MT-GBLUP) allows simultaneous prediction for genetically correlated traits, thereby enhancing accuracy for both low- and high-heritability traits and capturing complex relationships among traits, which are essential for improving overall crop performance^24,25^. In sorghum breeding, the use of ST-GBLUP has been more common^14,15,26^. However, there is a growing awareness of the benefits of MT-GBLUP, which has led to its increased adoption. By utilizing information from genetically correlated traits, MT-GBLUP significantly enhances prediction accuracy, especially for traits with low heritability that are often difficult to evaluate and improve using traditional breeding methods. Despite the advantages of MT-GBLUP, there is limited published research detailing its practical applications and outcomes specifically within sorghum breeding. While studies indicate its effectiveness in other crops^27^ and for certain traits^24,28^, comprehensive examples demonstrating its real-world application in sorghum are still scarce. Most existing literature tends to focus on single-trait predictions or theoretical evaluations of MT-GBLUP^29^.

Addressing the existing research gap is crucial because improving prediction accuracy through MT-GBLUP can significantly advance sorghum breeding programs. Enhanced methodologies can accelerate the development of sorghum varieties that possess desirable traits such as biofortification, drought resistance, pest tolerance, and increased yields—qualities that are becoming increasingly important due to climate change and a growing global population. Incorporating MT-GBLUP into breeding programs can enhance crop improvement, contributing to food security and sustainable agriculture. Additionally, applying GS can expedite the development of biofortified sorghum cultivars with higher iron (Fe) and zinc (Zn) content. GS allows for accurate selection in early generations, alleviating the costs and time associated with phenotyping advanced generation lines by enabling the identification of promising breeding lines at the seed stage.

We present our study on GS for grain Fe and Zn contents in sorghum using single-trait and multi-trait GBLUP models of genomic selection. In general, GS models exhibit comparable performance^14,24,30,31^, however, the GBLUP model of GS was chosen because it is considered easy to use and computationally efficient, which makes it a popular and practical method for routine genomic evaluations. Its simplicity and low computational requirements, compared to more complex Bayesian methods or deep learning models, are key reasons for its widespread adoption in breeding programs^32^. The objective of this study is to evaluate the effectiveness of multi-trait genomic selection (MT-GBLUP) compared to single-trait genomic selection (ST-GBLUP) in predicting grain iron (Fe) and zinc (Zn) concentrations in sorghum. By leveraging data from auxiliary agronomic traits, the research aims to enhance prediction accuracy and streamline breeding efforts, ultimately contributing to improved biofortification and nutrient density in sorghum. This study definitively establishes itself as the first to utilize GS in sorghum breeding for enhancing grain iron (Fe) and zinc (Zn) content. By rigorously comparing single-trait and multi-trait GS approaches, we confront the pressing challenges that breeders face in shortening breeding cycles and cutting testing costs. Our method effectively reduces both temporal and spatial replications without sacrificing the accuracy of data. This research not only elevates the nutritional value of sorghum but also revolutionizes the breeding process, solidifying its importance in advancing agricultural productivity and efficiency.

## Results

### Genotypic analysis

A total of 12,642 Quality Controlled SNPs were used to perform discriminant analysis of the principal components (DAPC), investigate the population structure of the studied minicore lines, and to perform single-trait and multi-trait based genomic prediction in the study using GBLUP model.

### Population structure analysis

**Fig. 2:**
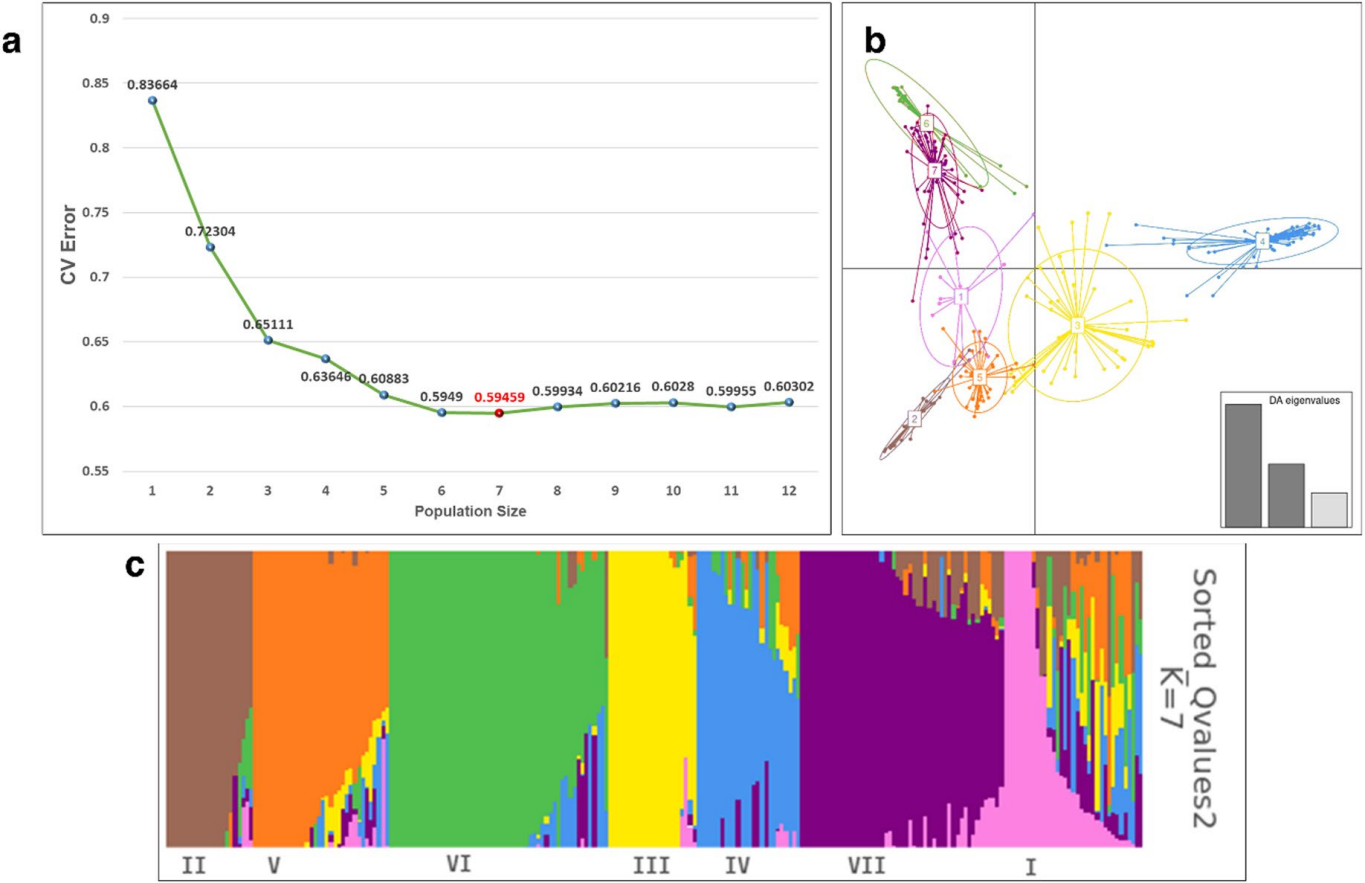
**(a)** Optimum population size estimation confirms K = 7 as the point with the lowest CV error; **(b)** Discriminant analysis of principal components (DAPC) representing the genetic structure of minicore population; **(c)** Population structure of minicore population (K = 7) using ADMIXTURE analysis.

The population structure was analysed by admixture-based model in ADMIXTURE software using SNP markers. The optimal number of sub-populations (K) was estimated using results of admixture analysis. The lowest cross-validation error (CV error) value was inferred at K = 7, suggesting the existence of seven sub-populations. DAPC analysis based on SNP markers also identified seven sub-populations (Fig. 2). Sub-population 1 was primarily composed of bicolor race accessions and its inter-racial hybrids, sub-population 2 and 3 mainly consisted of guinea race accessions and its inter-racial hybrids. Sub-population 4 was largely formed by kafir and caudatum races along with their inter-racial hybrids, while sub-population 5 predominantly comprised caudatum race accessions and its inter-racial hybrids. Sub-population 6 was mainly represented by inter-racial hybrids of the bicolor race, and sub-population 7 was dominated by durra race accessions and their inter-racial hybrids.

### Phenotypic analyses, line basis heritability, and genetic correlations among traits

**Fig. 3:**
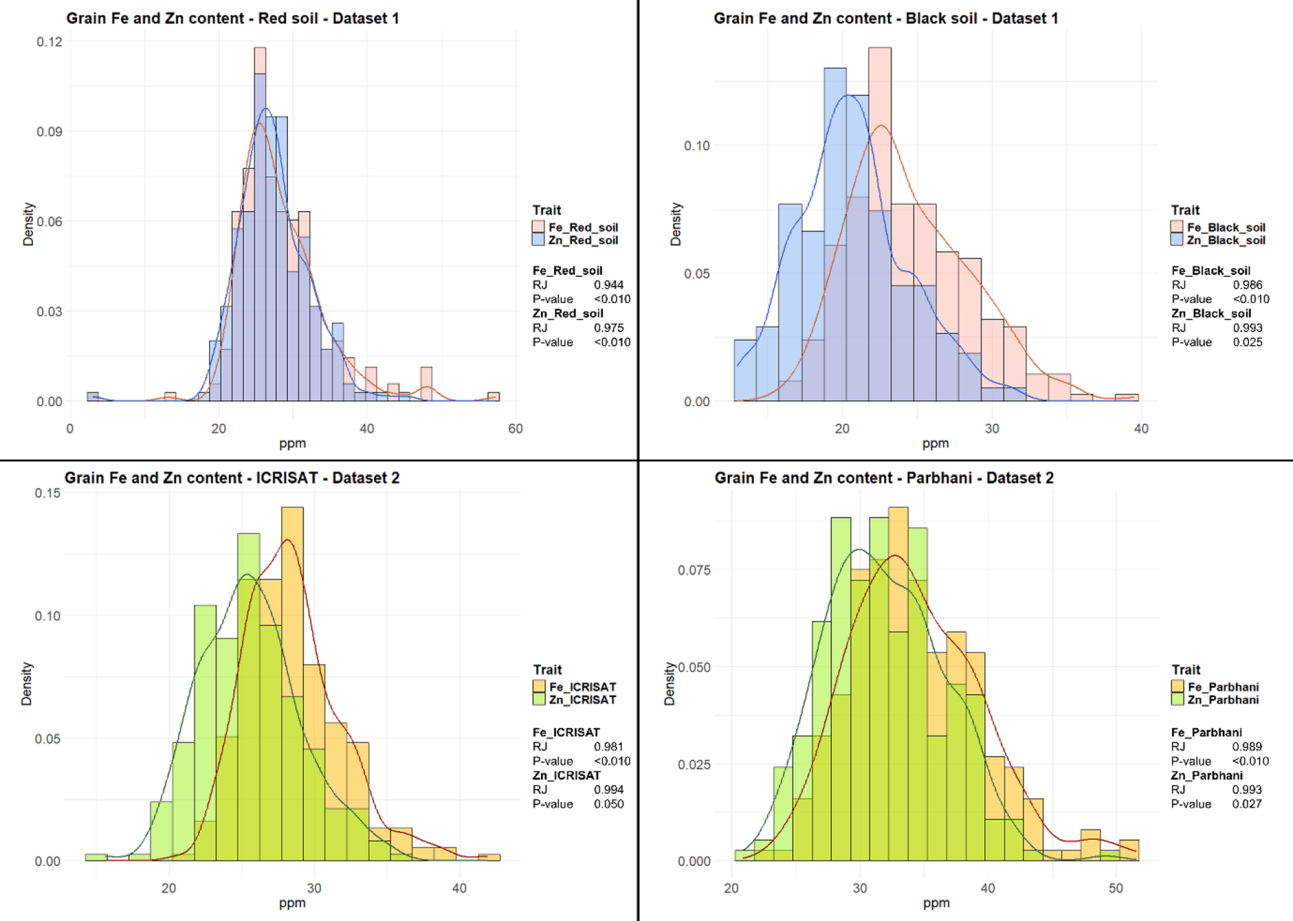
Graphical representation of grain iron and zinc frequency distributions for both datasets (DS1 and DS2).

The adjusted means estimated across environments in both datasets (DS1 and DS2) for grain Fe and Zn content are presented in Fig. 3. The frequency distribution of the phenotypic data followed an approximate normal distribution, as suggested by Shapiro-Wilks normality test^33^.

**Fig. 4:**
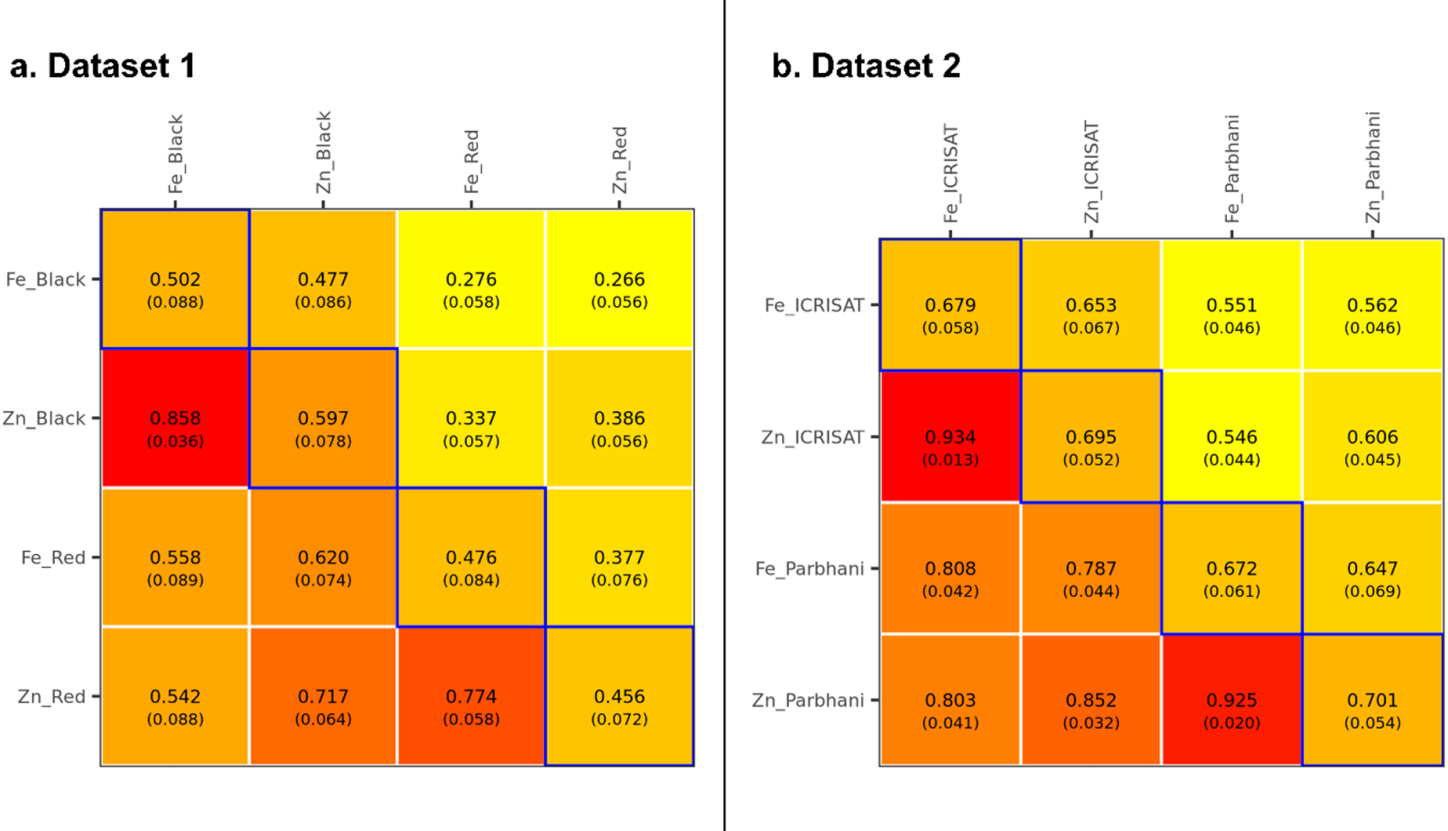
Genetic (co)variances (above the diagonal), genetic correlations (below the diagonal), and heritabilities (in the diagonal) of grain Fe and Zn content within Black and Red environments (Dataset 1, $\:\mathrm{n}=225$), and within ICRISAT and Parbhani environments (Dataset 2, $\:\mathrm{n}=244$). The estimates displayed are posterior means and posterior standard deviations (in parentheses).

**Fig. 5:**
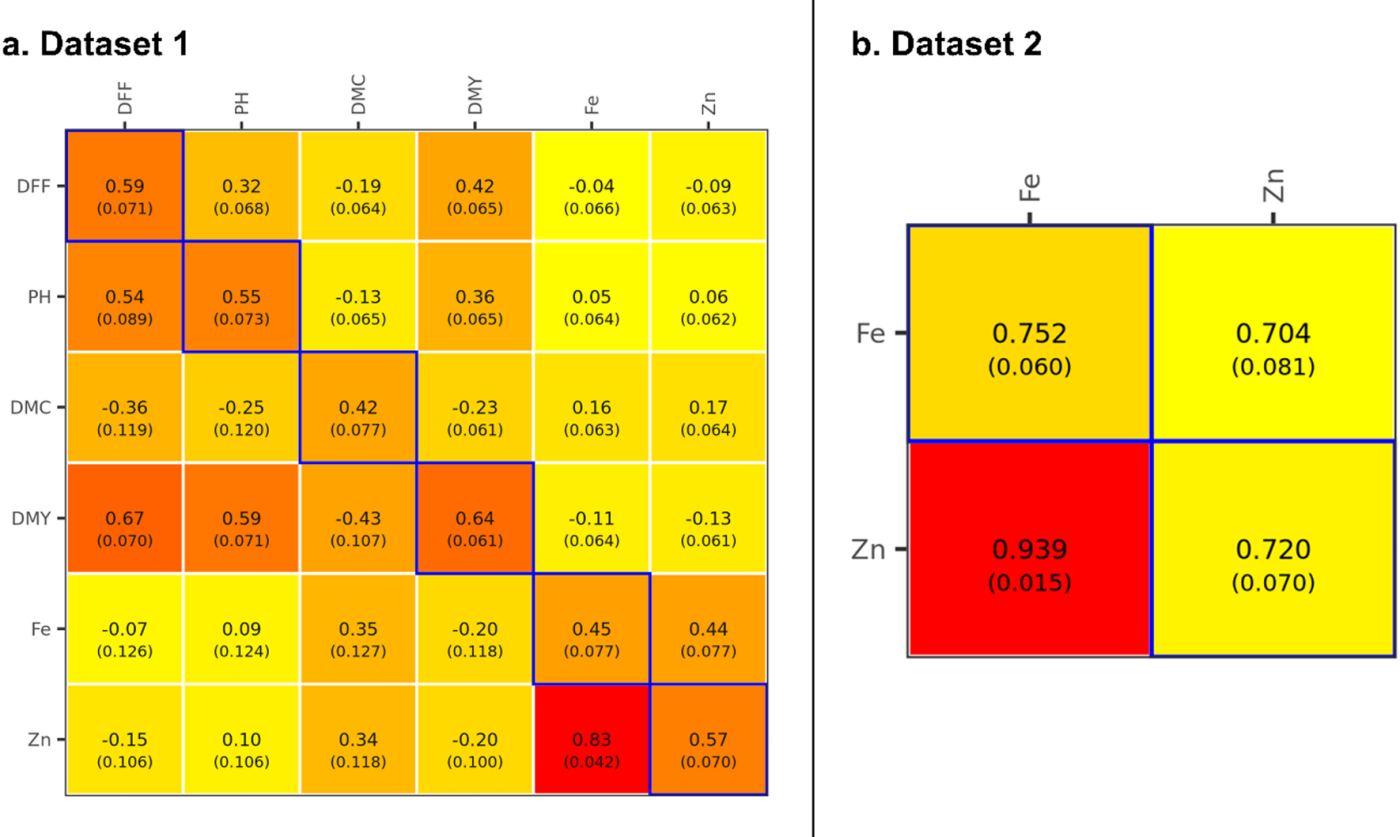
Genetic (co)variances (above the diagonal), genetic correlations (below the diagonal), and heritability (in the diagonal) of grain Fe and Zn, DFF, PH, DMC, and DMY across environments (Dataset 1, $\:\mathrm{n}=245$), and of grain Fe and Zn across environments (Dataset 2, $\:\mathrm{n}=245$). The estimates displayed are posterior means and posterior standard deviations (in parenthesis).

The within-environment line basis heritability values were moderate for grain Fe and Zn contents in DS1, ranging from 0.456 to 0.597 (Fig. 4a) and high in DS2 with range varying from 0.672 to 0.701 (Fig. 4b). The heritability obtained across environments in DS1 (two soil types at a single location and year i.e., Black soil and Red soil at ICRISAT) varied from moderate to high values for different traits under study with moderate heritability for grain Fe (0.45), grain Zn (0.57), days to 50% flowering (DFF) (0.59), plant height (PH) (0.55), dry mass fraction of the fresh material (DMC) (0.42) and high heritability for dry matter yield (DMY) (0.64) (Fig. 5a). While in DS2, the across-environments (two locations over years i.e., ICRISAT and Parbhani over three years) heritability was high for grain Fe (0.752) and Zn (0.720) content (Fig. 5b). The moderate to high heritability values suggest the involvement of genetic factors in most of the variation associated with these traits, was substantial.

The genetic correlation between grain Fe and Zn within-environments ranged from 0.542 to 0.858 in DS1 (Fig. 4a), and 0.787 to 0.934 in DS2 (Fig. 4b). Across-environments genetic correlations between different traits studied in DS1 viz., DFF, PH, DMC, DMY, grain Fe and Zn content are presented in Fig. 5a. The correlation coefficients ranged from − 0.43 to 0.83, with moderate positive correlation between DFF and DMY (0.67), and high positive correlation between grain Fe and Zn content (0.83). Similar high positive genetic correlation was observed between grain Fe and Zn content (0.939) in across-environments data of DS2 (Fig. 5b).

### Prediction accuracy (PA)

We used cross-validation scheme 2 (CV2) to assess the ability of genomic predictions for all traits under study. The CV2 scheme outperformed other cross-validation schemes in multi-trait GS models (*p* < 0.05) over single-trait GS model, according to Muvunyi et al.^34^. Phenotypic data of auxiliary traits were available in both training and testing sets in MT-GBLUP model of this scheme (CV2). The prediction accuracies are provided as average across 100 training-testing partitions of CV2 type scheme in both single-trait GBLUP (ST-GBLUP) and multi-trait GBLUP (MT-GBLUP) models. The ST-GBLUP model was selected as a baseline to compare the PA gain obtained with MT-GBLUP model.

**Table 1:** List of trait combinations investigated in the cross-validation 2 (CV2) scheme.

GS Model		Training set		Testing set	
	Dataset	Target trait	Auxiliary traits	Target trait	Auxiliary traits
Scenario 1*					
**ST-GBLUP**	**DS1**	Fe_Black	-	Fe_Black	-
		Zn_Black	-	Zn_Black	-
		Fe_Red	-	Fe_Red	-
		Zn_Red	-	Zn_Red	-
	**DS2**	Fe_ICRISAT	-	Fe_ICRISAT	-
		Zn_ICRISAT	-	Zn_ICRISAT	-
		Fe_Parbhani	-	Fe_Parbhani	-
		Zn_Parbhani	-	Zn_Parbhani	-
**MT-GBLUP**	**DS1**	Fe_Black	Zn_Black	Fe_Black	Zn_Black
			Fe_Red		Fe_Red
			Zn_Red		Zn_Red
		Zn_Black	Fe_Black	Zn_Black	Fe_Black
			Fe_Red		Fe_Red
			Zn_Red		Zn_Red
		Fe_Red	Fe_Black	Fe_Red	Fe_Black
			Zn_Black		Zn_Black
			Zn_Red		Zn_Red
		Zn_Red	Fe_Black	Zn_Red	Fe_Black
			Zn_Black		Zn_Black
			Fe_Red		Fe_Red
	**DS2**	Fe_ICRISAT	Zn_ICRISAT	Fe_ICRISAT	Zn_ICRISAT
			Fe_Parbhani		Fe_Parbhani
			Zn_Parbhani		Zn_Parbhani
		Zn_ICRISAT	Fe_ICRISAT	Zn_ICRISAT	Fe_ICRISAT
			Fe_Parbhani		Fe_Parbhani
			Zn_Parbhani		Zn_Parbhani
		Fe_Parbhani	Fe_ICRISAT	Fe_Parbhani	Fe_ICRISAT
			Zn_ICRISAT		Zn_ICRISAT
			Zn_Parbhani		Zn_Parbhani
		Zn_Parbhani	Fe_ICRISAT	Zn_Parbhani	Fe_ICRISAT
			Zn_ICRISAT		Zn_ICRISAT
			Fe_Parbhani		Fe_Parbhani
Scenario 2**					
**ST-GBLUP**	**DS1**	Fe	-	Fe	-
		Zn	-	Zn	-
	**DS2**	Fe	-	Fe	-
		Zn	-	Zn	-
**MT-GBLUP**	**DS1**	Fe	DFF	Fe	DFF
			PH		PH
			DMC		DMC
			DMY		DMY
			Zn		Zn
		Zn	DFF	Zn	DFF
			PH		PH
			DMC		DMC
			DMY		DMY
			Fe		Fe
	**DS2**	Fe	Zn	Fe	Zn
		Zn	Fe	Zn	Fe

*Scenario 1: Prediction accuracy of within-trait_environment combinations for grain Fe and Zn content in the ST-GBLUP method (using only target trait_environment) and MT-GBLUP method (using the target trait_environment and micronutrient auxiliary trait_environments).

**Scenario 2: Prediction accuracy of across-environments trait combinations for grain Fe and Zn content in the ST-GBLUP method (using only target traits) and MT-GBLUP method (using the target trait, agronomic, and micronutrient auxiliary traits in DS1 and only target trait and micronutrient auxiliary trait in DS2).

#### Scenario 1: PA gain for grain Fe and Zn content by MT-GBLUP using only micronutrient traits as auxiliary traits in datasets with restricted Spatial trials vs. numerous Spatial and Temporal trials

**Dataset 1 (DS1)**.

**Fig. 6:**
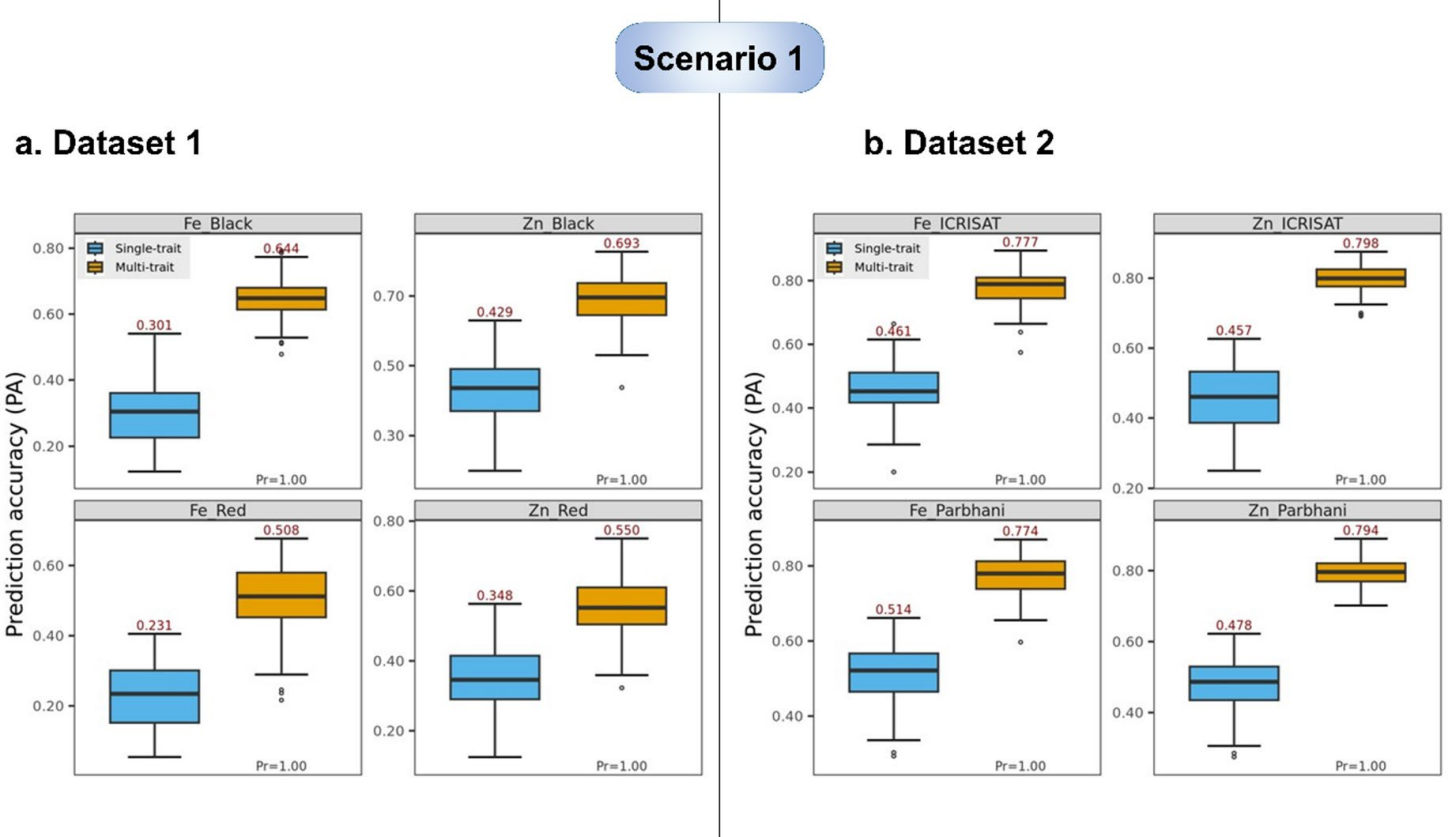
Prediction accuracy (PA, average across 100 training-testing partitions of a CV2-type scheme) achieved by the single-trait-environment GBLUP (Dataset 1: $\:{n}_{TS{T}_{j}}=66$, $\:{n}_{TR{N}_{j}}=159$. Dataset 2: $\:{n}_{TS{T}_{j}}=72$, $\:{n}_{TR{N}_{j}}=172$) and multi-trait-environment GBLUP (Dataset 1: $\:{n}_{TST}=264$, $\:{n}_{TRN}=636$. Dataset 2: $\:{n}_{TST}=288$, $\:{n}_{TRN}=688$) models for grain Fe and Zn within-environment adjusted means. *Pr*: Proportion of times that the PA of the multi-trait-environment GBLUP was higher than that of the single-trait-environment GBLUP.

In the DS1 with restricted spatial trials (Black and Red soil types) at a single location (ICRISAT) and year (Post rainy, 2023), the environment-wise (soil type wise) grain Fe and Zn content PA obtained by ST-GBLUP and MT-GBLUP were 0.301, 0.644 for Fe; 0.429, 0.693 for Zn in black soil, and 0.231, 0.508 for Fe; 0.348, 0.550 for Zn in Red soil respectively (Fig. 6a). Data of grain Fe and Zn content from both environments (Soil types) were utilised as auxiliary traits for estimation of PAs in the MT-GBLUP model (Table 1). In both environments (soil types), Zn had a relatively higher PA than Fe for both MT-GBLUP and ST-GBLUP models, with the former being superior.

**Dataset 2 (DS2)**.

In DS2 with numerous spatial (ICRISAT and Parbhani) and temporal (Post rainy 2020, 2021, 2022) trial combinations, the estimates of location-wise (pooled over years) PA using ST-GBLUP and MT-GBLUP were 0.461, 0.777 for Fe; 0.457, 0.798 for Zn at ICRISAT over years, and 0.514, 0.774 for Fe; 0.478, 0.794 for Zn at Parbhani over years, respectively (Fig. 6b). Grain Fe and Zn content data from both locations viz., ICRISAT and Parbhani over years were used as auxiliary traits for estimation of PAs of grain Fe and Zn content by MT-GBLUP model. PA for grain Fe and Zn contents were consistently comparable across models, but they were improved by using MT-GBLUP in DS2 as observed in DS1.

### Prediction accuracy gain in scenario 1

The MT-GBLUP model improved the environment-wise PA for grain Fe and Zn content in both datasets (DS1 and DS2) by using micronutrient traits as auxiliary traits in both training and testing sets (Table 1; Fig. 6). In DS1, with Fe and Zn exhibiting moderate heritability and moderately strong to strong genetic correlations (Fig. 4a), the PA gains were 0.343 for Fe, 0.264 for Zn in black soil, and 0.277 for Fe, 0.202 for Zn in red soil. In case of DS2, the PA gains were recorded as 0.316 for Fe, 0.341 for Zn in ICRISAT, and 0.260 for Fe, 0.316 for Zn in Parbhani (Fig. 6). High heritability and strong genetic correlations were reported for Fe and Zn in DS2 (Fig. 4b). No significant clear-cut difference was observed in PA gain for grain Fe and Zn content in DS1 with restricted spatial trials vs. DS2 with numerous spatial and temporal trials. However, the PA value was high in DS2 for grain Fe and Zn content when there was no difference in number of auxiliary traits used in MT-GBLUP of both datasets. This is supported by high heritability and strong genetic correlations between grain Fe and Zn content in case of DS2 based on data from numerous spatial and temporal trials.

#### Scenario 2: Across-environments PA gain for grain Fe and Zn content by MT-GBLUP in datasets with several auxiliary traits (agronomic and micronutrient traits) in restricted Spatial trials vs. few auxiliary traits (micronutrient traits) in numerous Spatial and Temporal trials

**Dataset 1 (DS1)**.

**Fig. 7:**
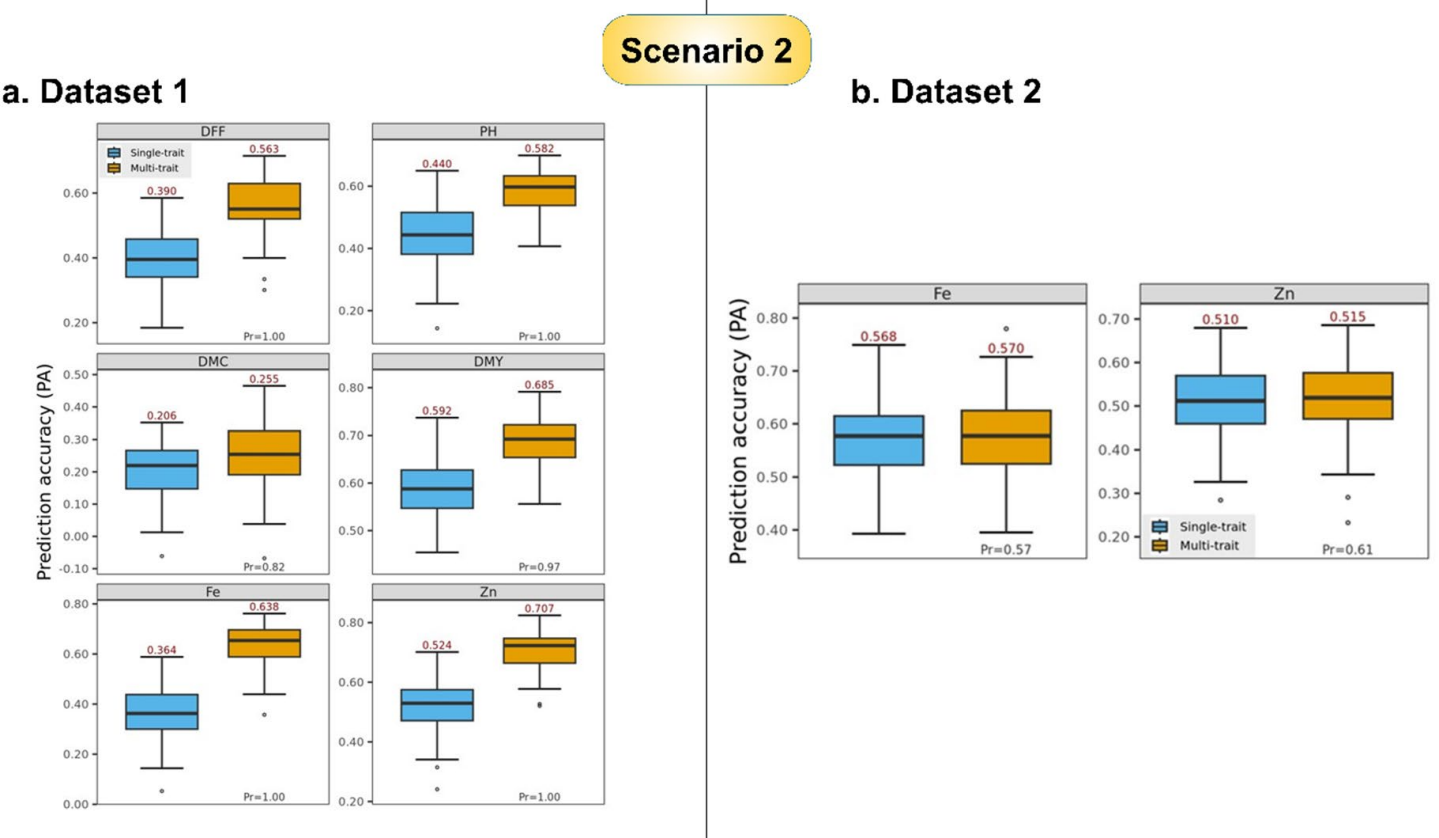
Prediction accuracy (PA, average across 100 training-testing partitions of a CV2-type scheme) achieved by the single-trait GBLUP (Dataset 1: $\:{n}_{TS{T}_{j}}=70$, $\:{n}_{TR{N}_{j}}=174$. Dataset 2: $\:{n}_{TS{T}_{j}}=74$, $\:{n}_{TR{N}_{j}}=171$) and multi-trait GBLUP (Dataset 1: $\:{n}_{TST}=420$, $\:{n}_{TRN}=\mathrm{1,044}$. Dataset 2: $\:{n}_{TST}=148$, $\:{n}_{TRN}=342$) models, for grain Fe and Zn, PH, DMC, and DMY (Dataset 1); and for grain Fe and Zn (Dataset 2) across-environments adjusted means. *Pr*: Proportion of times that the PA of the multi-trait-environment GBLUP was higher than that of the single-trait-environment GBLUP.

This dataset consisted of data on agronomic traits (DFF, PH, DMY, DMC) in addition to grain Fe and Zn content in restricted spatial trials. PA in across environments ST-GBLUP was 0.364 for grain Fe and 0.524 for Zn content. High correlation was recorded between grain Fe and Zn content, and low correlation was noted between micronutrient traits (Fe and Zn) and agronomic traits (DFF, PH, DMY, DMC) (Fig. 5a). When MT-GBLUP model was used to predict the across-environments PA for grain Fe and Zn content using data of all other traits (agronomic traits and grain micronutrients) as auxiliary traits (Table 1), the PAs improved to 0.638 for Fe and 0.707 for Zn content (Fig. 7a). Similar trend of PA improvement was recorded for agronomic traits with MT-GBLUP method over ST-GBLUP, with PA gains of 0.173 (DFF), 0.142 (PH), 0.049 (DMC) and 0.093 (DMY).

**Dataset 2 (DS2)**.

This dataset has information only on grain Fe and Zn content from numerous spatial and temporal trial combinations. The across-environments PAs obtained using ST-GBLUP for Fe was 0.568 and for Zn was 0.510. High genetic correlation was recorded between these two traits. The PAs obtained by MT-GBLUP on using these two traits (Fe and Zn) data as auxiliary traits (Table 1) were 0.570 (Fe) and 0.515 (Zn) (Fig. 7b).

### Prediction accuracy gain in scenario 2

In terms of PA gain, we observed an improvement in PA in both datasets for grain Fe and Zn content using MT-GBLUP in scenario 2 with PA gain values of 0.274 and 0.183 in DS1, and 0.002 and 0.005 in DS2 respectively (Fig. 7). The DS1 with data on several auxiliary traits over restricted spatial trials presented higher estimates of PA values and PA gains by MT-GBLUP model. The disparity in the number of auxiliary traits involved in DS1 and DS2 had great effect on PA. The MT-GBLUP model in DS1 exhibited greater gains for traits showing moderate heritability and was efficient in utilizing the borrowed information from both high and low correlated traits. While in DS2 where information was borrowed from only one highly correlated trait, the PA gain was minimal.

## Discussion

Multi-environment trials (MET) are critical in any breeding program for assessing genotype performance and stability to guide the promotion of superior materials. The early-generation recycling of the parents and large number of breeding materials to evaluate in multiple locations, limited time to test the genotypes over the years, and its associated costs are among the major challenges faced by breeders for effective phenotypic selection. These trials are also expensive, with considerable cost associated with each replicate^35^. Genomic selection (GS) is a strategy that addresses these issues by assisting the breeder in effective selection of superior genotypes based on GEBVs of unphenotyped genotypes. It saves time and cost compared to METs if models are trained using good-quality genotypic and phenotypic data and show good PA. Using GS approaches would accelerate genetic gains in the production of nutrient-enhanced sorghum cultivars, as these traits are particularly difficult and expensive to measure. The GS enhances genetic gain by unit time and cost, particularly in virtue of the intercrosses driven by the GEBV, which shortens the breeding cycle^11^. The GEBV refers to the statistical prediction of an individual’s genetic merit (breeding value) based on its whole-genome molecular marker information. The GEBV therefore, estimates the genetic potential of an individual for a trait of interest by accounting for the combined effects (beta coefficients) of multiple markers across the entire genome. It is now acknowledged that selection based on GEBVs and accurate phenotyping is the obligatory pathway to sustainable genetic gain^19,20^.

The improvement in genomic PA is crucial before selection by assessing prediction model performance through cross-validation. This is because GS accuracy provides an estimate of selection accuracy, which is directly linked to selection response (R), also referred to as genetic gain^19^. GS enhances both accuracy and genetic gain in breeding programs. Vice versa, higher GS accuracy leads to greater genetic gain, which is the increase in performance per unit of time due to selection. Ultimately, GS enables breeders to make more precise selections, resulting in faster and more efficient improvements in desirable traits. Single-trait GS models remain popular in GS, however, they have a low PA for complex traits because of their complicated genetic architecture, and the inherent poor parameter estimate and the selection biases due to multiple independent modelling processes. Multi-trait GS models have been proposed in recent years to improve the PA of complex traits when auxiliary trait phenotypic data is available^36^. Multi-trait GS has been applied in sorghum for the prediction of biomass yield^17,28^, grain yield^24^ and antioxidant production^14^. Genomic selection was used to determine grain Fe and Zn concentration in wheat^37–39^, rice^34^, pea^27^, common beans^40^, and yellow dry beans^41^.

The sorghum minicore collection maintained at gene bank of ICRISAT was used for this study to compare the PA of ST-GBLUP and MT-GBLUP models of GS as it holds a full base of genetic diversity of sorghum biofortification useful in breeding for the enhanced grain Fe and Zn content. The experiments and data were presented to mimic the breeder’s need to complete the breeding cycle in the shortest time without jeopardizing the selection accuracy. This aligns with the current recommendations from breeding institutions in order to boost the genetic gain, a high-level of key performance indicator (KPI) of breeding programs. The breeder has limited time for recycling materials and replicating the material over time (i.e., seasons or years), but can potentially replicate more times over space in a few seasons for efficient genotype selection with better PA. This situation is particularly observed in the early generations (or) stage 1 trials, where a large number of materials is involved.

We intentionally selected two different soil types (Black soil and Red soil) as different environmental conditions over space at a single location in DS1. The choice of space in DS1 was crucial as maintaining different sowing dates or considering stress and non-stress conditions as two distinct environments over space - a common practice in breeding - would deviate from maintaining the properties of the target environment conditions that are otherwise necessary for obtaining unbiased prediction accuracies. Reports of earlier studies suggest that unfavourable (stress) conditions affect estimates of heritability and subsequently affect the estimates of PA^42^^[,43^.

In scenario 1, the within-environment PA gain for grain Fe and Zn content by MT-GBLUP over ST-GBLUP was tested by including only high-correlated auxiliary traits (micronutrient traits) in MT-GBLUP model in restricted spatial trials (DS1) vs. more spatial and temporal trials (DS2). Considerable PA gain was reported in both datasets by using MT-GBLUP model. The PA gain and PA values were higher for DS2 with more trial replicates, reflected from the high heritability pattern of the traits in the specific environments under study when only micronutrient traits were considered as auxiliary traits in both datasets. In scenario 2, across-environments PA gain for grain Fe and Zn content by MT-GBLUP over ST-GBLUP was tested by including several low-, moderate- and high- correlated auxiliary traits (agronomic traits - DFF, PH, DMY, DMC and micronutrient traits) in restricted spatial trials (DS1) vs. only high-correlated auxiliary traits (micronutrient traits) in numerous spatial and temporal trials (DS2). The MT-GBLUP model outperformed the ST-GBLUP model in both datasets for PA values of Fe and Zn. However, the PA gain achieved was greater in DS1, where information was borrowed from numerous auxiliary traits with varying correlations (low, moderate, and high correlated traits), than in DS2, where information was borrowed from only one highly correlated trait. In this study, we tried and succeeded to explore the potential for inclusion of easily scorable agronomic auxiliary traits in the training and testing set in MT-GBLUP model of GS for improved genomic prediction of the target traits without extensive spatial or temporal replications.

Similar findings of improved PA for selection of primary traits by including simple auxiliary traits (easy to score and cost effective) with low and moderate correlation were reported for end-use quality traits^36^, yield traits^44^, deoxynivalenol accumulation resulting from fusarium head blight^45^ in wheat, diameter at breast height in Shining gum^46^ and grain quality traits in barley^47^.

This research has limitations, including computational complexity, trait correlation, and data requirements. While MT-GBLUP offers better prediction accuracy, it demands more computational resources than Single-Trait GBLUP, resulting in longer processing times and the need for advanced infrastructure, especially with large datasets. MT-GBLUP relies on correlated traits; inaccuracies can arise if the correlation is misestimated or if traits interact in complex ways. Additionally, high-quality datasets across multiple environments are crucial for its effectiveness. Sparse or unbalanced data can reduce predictive accuracy, and missing data can complicate analyses. These factors should be considered when implementing MT-GBLUP in genomic selection studies.

Our research findings, however, indicate that the accuracy of genomic selection predictions for grain iron (Fe) and zinc (Zn) content in sorghum can be enhanced by using a multi-trait GBLUP model (MT-GBLUP). These results are particularly relevant for plant breeders, as they demonstrate that the MT-GBLUP model improves prediction accuracy for target traits by incorporating auxiliary target (micronutrients) and non-target (agronomic) data, especially in restricted spatial trials. Implementing the MT-GBLUP model is expected to benefit breeding programs, particularly where traits are often correlated, as it allows for the exploitation of these correlations to enhance overall selection accuracy. MT-GBLUP is an integrative approach that will lead to faster genetic gain over time, as breeders can select for traits that benefit from positive genetic correlations, thereby maximizing the benefits across traits.

## Methods

### Experimental material and field trials

**Fig. 8:**
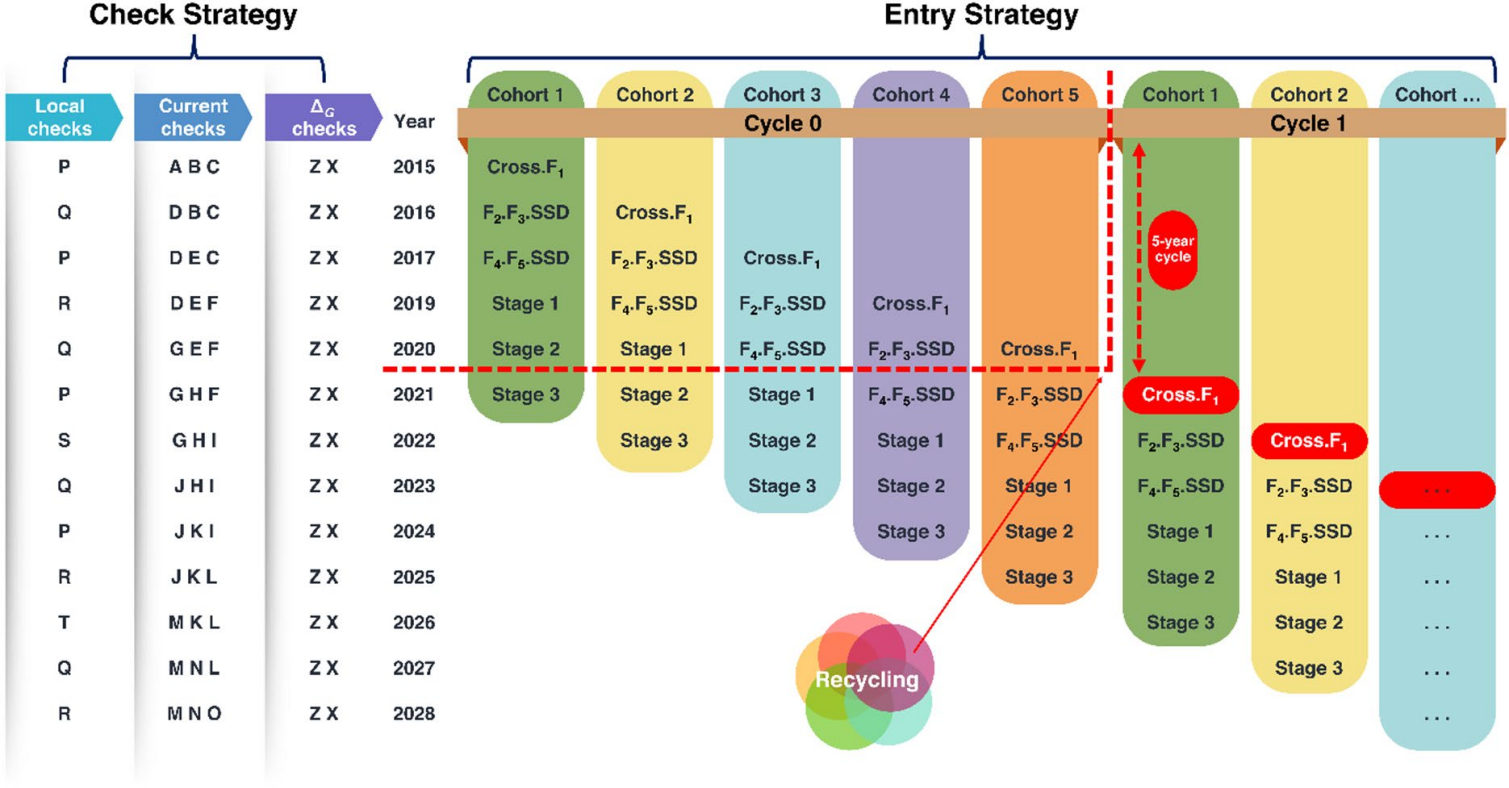
GS-driven five-year recycling for population improvement. Recycling is informed by GS-predicted GEBVs of unphenotyped materials. ∆_G_: genetic gain; SSD: single seed descent selection, F_1_: first filial progeny, stage 1: MET stage 1, cohort: selection stream from intercrosses in a crossing block for a unique market segment. (Picture adapted from Excellence in Breeding (EiB) Platform, CGIAR^48^.

All the trials were conducted in open fields using sorghum accessions from the minicore collection maintained at the Genebank of the International Crops Research Institute for the Semi-Arid Tropics (ICRISAT, Patancheru). These accessions were allocated into two trial datasets - dataset 1 (DS1) and dataset 2 (DS2) (Table 2). DS1 and DS2 mimic the breeders’ working constraints wherein, on the one hand, they can face limited resources and hence test the sorghum materials in one season, one or a few locations and a few (e.g., 2) environments but collect data on many traits (DS1). This is similar to the current CGIAR-recommended, GS-driven recycling strategy at the first stage of multi-environment trials (METs) during population improvement. GS-derived GEBVs are used to inform the recycling of the parents, while the stage 1 METs phenotypic data are used to identify the products to advance to stage 2 METs (Fig. 8), applying appropriate selection intensity^48^. On the other hand, in case of enough resources, breeders can test the materials under several (e.g., 3) seasons and locations (e.g., 2) and collect data only on the target traits (DS2). In both scenarios, the resources are used efficiently.

**Table 2:** Latitude and longitude data of experimental sites used in the study.

Dataset 1		Dataset 2	
Environment	Latitude, Longitude	Environment	Latitude, Longitude
Red soil - ICRISAT, 2023	17.50885, 78.28082	ICRISAT, 2020	17.51281, 78.26759
Black soil - ICRISAT, 2023	17.51074, 78.26773	Parbhani, 2020	19.25111, 76.76635
		ICRISAT, 2021	17.48248, 78.26122
		Parbhani, 2021	19.24088, 76.78543
		ICRISAT, 2022	17.51303, 78.26586
		Parbhani, 2022	19.24088, 76.78543

**Dataset 1 (DS1)**.

The dataset 1 consisted of 252 genotypes (242 minicore accessions + 10 checks), evaluated in restricted spatial trials i.e., two different soil types (black soil and red soil) at a single location - ICRISAT, Patancheru and a single year i.e., post-rainy season of 2023 (Table 2). The two soil types represented two environments in DS1. The plots in each environment were laid out in an alpha lattice design with 21 incomplete blocks and two replications.

**Dataset 2 (DS2)**.

The dataset 2 consisted of 250 genotypes (242 minicore accessions + 8 checks), evaluated in several locations and seasons i.e., at two different locations: International Crops Research Institute for the Semi-Arid Tropics (ICRISAT), Patancheru and Vasantrao Naik Marathwada Krishi Vidyapeeth, Parbhani in post rainy season of 2020, 2021, and 2022 (Table 2). Six different environments were formed as a year-location combination in DS2. Plots in these trials were laid out in an alpha lattice design with 25 incomplete blocks and two replications.

In both datasets, each genotype was raised in a plot of a single row of 4 m length, with a row-to-row spacing of 60 cm and plant-to-plant spacing of 15 cm within a row. In our experimental fields, the available Fe and Zn content in soil was sufficient for plants to grow and develop normally, surpassing the minimum needed amounts of 4.50 to 6.50 mg kg^− 1^ Fe and 0.6 to 0.9 mg kg^− 1^ Zn. All the trials were conducted in the post-rainy (also known as Rabi) season following standard agronomic practices to ensure proper growth and good quality of the seed.

### Phenotypic data

As the grains reached the physiological maturity stage, the panicles were harvested to determine the Fe and Zn contents in both datasets. Grain sampling was done as described in previous works^4^. The grain Fe and Zn concentration was determined using X-ray fluorescence spectrometry (XRF) (Make: Hitachi High-Tech, Japan; model: X-Supreme8000), which is a calibrated, efficient, non-destructive, and cost-effective analytical method.

The DS1 contains, in addition, the agronomic data on days to 50% flowering (DFF), plant height (PH) (cm), dry matter yield (DMY) (Kg ha^− 1^), and dry mass fraction of the fresh material (DMC) (%). The plant height was measured a week before harvest as the mean of the elementary plot using a 5 m aluminium telescopic gauge composed of 5 rods, with mm scale in front, and geodetic scale on the back (METRICA, Made to measure, Italy). The whole aboveground matter of the entire plot was manually chopped and scored for fresh biomass yield (Kg plot^− 1^), which was converted into fresh biomass yield (Kg ha^− 1^). A sample from the fresh biomass was taken from each plot and was immediately noted for fresh weight, and after the sample was oven dried at 80˚C to constant weight, dry weight was noted. The dry mass fraction of the fresh material in percentage was calculated as DMC% = (sample dry weight/sample fresh weight) × 100. The dry biomass yield in kg ha^− 1^ was calculated as DMY (in Kg ha^− 1^) = Fresh biomass yield (Kg ha^− 1^) × Dry mass fraction of fresh material (%).

### Genotypic data

#### Isolation of DNA and genotyping

The procedure followed in DNA isolation and genotyping was amply described in previous work^4^. The genotyping-by-sequencing (GBS) single nucleotide polymorphisms (SNPs) data produced by ICRISAT was used in this work. The genotypic information consisted of 12,642 quality controlled SNPs coded as 0, 1, 2, representing the number of copies of the reference allele for 242 minicore genotypes. These SNPs passed the filters, including minor allele frequency (MAF) > 0.05 and missing data frequency < 0.15. Remaining missing values were imputed as the marginal mean, i.e., the sample mean of observed genotypes at a given locus.

### Statistical analysis of phenotypic data

#### Adjusted means

To estimate the adjusted means, all phenotypes were adjusted by experimental design as follows:

##### Within-environment

the following equation was solved.

$${Y_{ijk}} = \mu + Re{p_i} + Block:Re{p_{j\left( i \right)}} + Ge{n_k} + {_{ijk}}$$

Where $\:{Y}_{ijk}$ is the value of the observed trait for *k*^*th*^ genotype in the *j*^*th*^ block within *i*^*th*^ replicate, $\:\mu\:$ is the overall mean, $\:Re{p}_{i}$ is the effect of *i*^*th*^ replicate, $\:Block:Re{p}_{j\left(i\right)}$ is the effect of *j*^*th*^ block within the *i*^*th*^ replicate, $\:Ge{n}_{k}$ is the fixed effect of the *k*^*th*^ genotype, and $\:{\epsilon}_{ijk}$ is the experimental error.

All factors or terms in the analysis, except for “Genotype,” were treated as random effects and assumed to be independent and identically distributed (IID) from a normal distribution with mean zero and their corresponding variance.

The genotype term was considered as a fixed effect from which the least squared means were taken as the adjusted phenotype.

##### Across-environments

the following equation was solved.

$${Y_{ijkl}} = \mu \: + En{v_l} + Rep:En{v_{i\left( l \right)}} + Block:Rep:En{v_{j\left( {il} \right)}} + Ge{n_k} + Gen:En{v_{k\left( l \right)}} + {\varepsilon _{ijkl}}$$

Where $\:{Y}_{ijkl}$ is the value of the observed trait for *k*^*th*^ genotype in the *j*^*th*^ block within *i*^*th*^ replicate of *l*^*th*^ environment, $\:\mu\:$ is the overall mean, $\:En{v}_{l}$ is the effect of the *l*^*th*^ environment, $\:Rep:En{v}_{i\left(l\right)}$ is the effect of *i*^*th*^ replicate within *l*^*th*^ environment, $\:Block:Rep:En{v}_{j\left(il\right)}$is the effect of *j*^*th*^ block within the *i*^*th*^ replicate of *l*^*th*^ environment, $\:Ge{n}_{k}$ is the fixed effect of the *k*^*th*^ genotype, $\:Gen:En{v}_{k\left(l\right)}$ is the effect of *k*^*th*^ genotype within *l*^*th*^ environment, and $\:{\epsilon}_{ijkl}$ is the experimental error. For the dataset 2, the environments were represented by location × year combinations. It is a standard practice to model “location × year” as a distinct environment in mixed model equations for genomic selection, as this allows for capturing genotype-by-environment (G×E) interactions, which is crucial for accurate prediction of breeding values. While a specific year cannot be relived, the combination of location and year represents the unique environmental conditions at that time and treating it as an environment allows the model to learn how genotypes perform differently under those specific circumstances^49^.

Likewise, all terms except Genotype, were considered as random effects assumed to be IID Normal distributed. The genotype term was considered as a fixed effect from which least squared means were taken as the adjusted phenotype.

For the DS2, the within-environment adjustment was done across years, so we used the across-environment model, replacing the environment term with the year.

For the within-environment analysis, Fe and Zn content data were compiled for all 245 genotyped lines in both datasets, and lines with missing values for any one of the traits were excluded, and the number of usable lines in the phenotypic matrix was reduced accordingly. The adjusted phenotypic matrix contains 225 lines and 4 trait-environment combinations (Fe-Black, Zn-Black, Fe-Red, and Zn-Red) for the DS1; and 244 lines and 4 trait-environment combinations (Fe-ICRISAT, Zn-ICRISAT, Fe-Parbhani, and Zn-Parbhani) for the DS2.

For the across-environments analysis, the adjusted phenotypic matrix contains 245 lines and 6 traits (Fe, Zn, DFF, PH, DMC, and DMY) in DS1; and 245 lines and 2 traits (Fe and Zn) in DS2.

**Genomic prediction models**:

***Single-trait GBLUP***. The response (adjusted phenotypes) $\:\boldsymbol{y}=({y}_{1},\dots\:,{y}_{n}){\prime\:}$ was modeled as

$$\:\:{y_i} = \mu \: + {u_i} + {\:_i}$$.

The genetic values $\:\boldsymbol{u}=({u}_{1},\dots\:,{u}_{n}){\prime\:}$ are assumed ${u}{\sim}MVN\left( {0,\sigma _u^2G} \right)$ where the genomic relationship matrix (covariance matrix, GRM) is obtained as $\:\boldsymbol{G}=\frac{\boldsymbol{Z}{\boldsymbol{Z}}^{\boldsymbol{{\prime\:}}}}{trace\left(\boldsymbol{Z}{\boldsymbol{Z}}^{\boldsymbol{{\prime\:}}}\right)/n}$ with $\:\boldsymbol{Z}$ being the matrix of centered SNPs (lines in rows, SNPs in columns). The error terms $\:\boldsymbol{\epsilon\:}=({\epsilon\:}_{1},\dots\:,{\epsilon\:}_{n}){\prime\:}$ are assumed $\:\boldsymbol{\epsilon\:}{\sim}MVN(0,{\sigma\:}_{e}^{2}\boldsymbol{I})$.

**Multi-trait GBLUP**. Several responses vectors $\:{\boldsymbol{y}}_{1},{\boldsymbol{y}}_{2},\dots\:,{\boldsymbol{y}}_{q}$ are analyzed together as a single response $\:\boldsymbol{y}=({\boldsymbol{y}}_{1}^{{\prime\:}},{\boldsymbol{y}}_{2}^{{\prime\:}},\dots\:,{\boldsymbol{y}}_{q}^{{\prime\:}}){\prime\:}\:$,

$$y = \mu \: + u + \epsilon$$

where $\:\boldsymbol{u}=({\boldsymbol{u}}_{1}^{{\prime\:}},\dots\:,{\boldsymbol{u}}_{q}^{{\prime\:}}){\prime\:}{\sim}MVN(0,\boldsymbol{K}\otimes\:\boldsymbol{G})$ and $\:\boldsymbol{\epsilon\:}=({\boldsymbol{\epsilon\:}}_{1}^{{\prime\:}},\dots\:,{\boldsymbol{\epsilon\:}}_{q}^{{\prime\:}}){\prime\:}{\sim}MVN(0,\boldsymbol{R}\otimes\:\boldsymbol{I})$. Here $\:\boldsymbol{K}$ is the genetic variance-covariance matrix between traits, $\:\boldsymbol{R}$ is the residual variance-covariance matrix between traits, and $\:\otimes\:$ is the Kronecker product operator, which constructs the block matrix representing the combined genetic covariance for all individuals across all traits. The combination of the respective matrices through the Kronecker product resulted in a covariance matrix that captures the relationships between the effects due to both K and G, on the one hand, and R and I on the other. This structure was used in our models to accommodate multiple traits and/or environments, allowing for the analysis of complex genetic architectures in this work.

**Heritability**. Line-basis heritability was calculated for each trait within- and across-environments as $\:{h}^{2}={\sigma\:}_{u}^{2}/({\sigma\:}_{u}^{2}+{\sigma\:}_{e}^{2})$, and these estimates were extracted from the diagonal of the matrix $\:\boldsymbol{K}/(\boldsymbol{K}+\boldsymbol{R})$.

### Cross-validation of the GS models and prediction accuracy

To evaluate the prediction accuracy (PA) of the single-trait and multi-trait (or multi-environment) models, we performed prediction analysis using training-testing data partitions. We sampled the testing datasets using the cross-validation (CV) scheme CV2 described in Burgueño et al.^50^ and Jarquin et al.^51^, in which the goal is to predict the performance of lines that have been evaluated in some but not all the environments. Specifically, for each training-testing partition we iteratively sampled at random one (from 1 to $\:n$) line to be assigned to the testing set in only two (from a total of $\:{n}_{\mathrm{e}\mathrm{n}\mathrm{v}}$) environments, we continued sampling lines to be assigned to the testing set in different pairs of environments, such that, all the possible combinations of two taken from $\:{n}_{\mathrm{e}\mathrm{n}\mathrm{v}}$ environments were equally represented in the total testing set, and until ~ 30% of the $\:n$ lines within each environment was in the testing set ($\:{n}_{TS{T}_{j}}$). Therefore, the total number of data points forming each testing set was $\:{n}_{TST}={n}_{TS{T}_{j}}\times\:\:{n}_{env}$ phenotypic records. The total training dataset included the remaining $\:{n}_{TRN}={n}_{{TRN}_{j}}\times\:\:{n}_{\mathrm{e}\mathrm{n}\mathrm{v}}$ records where $\:{n}_{{TRN}_{j}}$ are the remaining ~ 70% within-environment lines not assigned to the testing set.

The single-trait GBLUP was implemented training the model using the $\:{n}_{TR{N}_{j}}$ records at the $\:{j}^{th}$ trait (and environment) to predict the $\:{n}_{TS{T}_{j}}$ testing records within the same environment. The multi-trait (within- or across environments) GBLUP was implemented, training the model using all the $\:{n}_{TRN}$ records together to predict all the $\:{n}_{TST}$ testing records. In this work, the MT-GBLUP operated by leveraging genetic information to predict the performance of multiple traits, and it functioned in both within-environment and across-environment contexts. In the context of a single environment, MT-GBLUP made use of the covariance among different traits to improve prediction accuracy. Across Environments, the MT-GBLUP took into account the interaction between genotypes and environments (G×E interaction). The ST-GBLUP, on the other hand, operates by modeling the genetic values of a phenotype based on genomic information. In a within-environment context, the ST-GBLUP focuses on estimating the genetic values of traits while accounting for specific environmental effects and variations observed within that particular environment. In contrast, the across-environment ST-GBLUP aimed to generalize the genetic value estimates for a trait by incorporating data from multiple environments as described in Burgueño et al.^50^.

Prediction accuracy (PA) was assessed by correlating observed and predicted values in the testing set, i.e., $\:cor({\boldsymbol{y}}_{{TST}_{j}},{\widehat{\boldsymbol{y}}}_{{TST}_{j}})$. These correlations were calculated within environment; specifically, for each environment, we estimated the accuracies using $\:{n}_{{\mathrm{T}\mathrm{S}\mathrm{T}}_{j}}$ per environment. In total, we performed 100 training-testing partitions, and the results presented correspond to averages across such partitions. During the cross-validation, we used the same 100 folds for both approaches to ensure a fair comparison. This means splitting the dataset into the same training and validation sets for each model. That is, the same 100-fold was applied to both the single-trait and multi-trait models. This consistency was critical for a valid comparison of their predictive performances.

### Software

All analyses were implemented in R software (R Core Team, 2024). Linear models for phenotypic adjustment were fitted using function “lmer” of the lme4 R-package^52^. The single-trait GBLUP models were fitted using the “fitBLUP” function of the SFSI R-package^53^. The multi-trait (or multi-environment) analyses were implemented using the “Multitrait” function of the BGLR R-package^54^. The genetic structure of population was analyzed using admixture analysis with “ADMIXTURE” software^55^ and population structure was visualized using “pophelper” shiny R package^56^. Discriminant Analysis of Principal Components (DAPC) was performed and visualized in R software using “adegenet”, “ggplot2”, and “vcfR” packages^57–60^.

## Data Availability

All necessary data is made available within the paper. The datasets generated and/or analysed during the current study are available in the “Figshare” repository. And can be found at https://doi.org/10.6084/m9.figshare.29827457.v1.
